# The experiences of people living with dementia and their care partners participating in an online therapeutic songwriting program

**DOI:** 10.1177/14713012231224069

**Published:** 2023-12-22

**Authors:** Imogen Clark, Neha Christopher, Phoebe Stretton-Smith, Kathleen Lawson

**Affiliations:** Faulty of Fine Arts and Music, 170539 University of Melbourne, Australia; 170539 University of Melbourne, Australia

**Keywords:** therapeutic songwriting, music therapy, dementia, care partner, telehealth

## Abstract

**Background and Aim:**

Despite the impact of dementia on close care relationships, accessible therapeutic services for people living with dementia and their care partners remain scarce. Further, there is an increasing demand for online services, highlighting the need for ongoing telehealth research. This study aimed to explore the experiences of people living with dementia and their informal and formal care partners following participation in a 10-week online therapeutic songwriting program.

**Methods:**

The songwriting program included four duo and six group sessions facilitated by a music therapist. Post-program semi-structured interviews were analysed using thematic analysis.

**Findings:**

Nine people with dementia and six care partners (nine duos) participated in the program. Five duos were in spousal relationships and four people with dementia participated with a formal care partner. Participants attended an average of four duo sessions and three group sessions. Six people with dementia and five care partners participated in post-program interviews. Four themes were developed: (1) No one else does this; (2) It’s all about us as people; (3) After the sessions we’d come out buzzing; and (4) The journey was as important as the product.

**Conclusions:**

Participants highlighted songwriting as a unique opportunity to connect with music, experience welcomed challenges, and spend ‘real time’ together. The program drew on participants’ lived experience and promoted connection with others, resulting in feelings of enjoyment, enhanced mood, and achievement. Participants valued both the songwriting process and song product, emphasising the importance of sensitive and skilful facilitation. Our findings suggest that these distinct benefits were not available through other support services. Further, online songwriting is a viable option for people living with dementia and their care partners where in-person sessions are not available or accessible.

## Introduction

The increasing global prevalence of dementia is a major concern impacting individuals, families, and society across personal, social, and economic domains (WHO, 2023). Dementia can affect a person’s memory, cognition, speech, movement, behaviour, and personality (Leung et al., 2021), and is recognised as a leading cause of disability, dependency, and death among older people (WHO, 2023). Further, dementia not only impacts the wellbeing of people with a diagnosis, but those who care for them, in turn influencing interpersonal relationships (Livingston et al., 2020; Rippon et al., 2020). Typically, care partner relationships are long-lasting, dynamic, and can be complex, requiring sensitive person-centred approaches which recognise both members of the partnership, whether informal spousal/family (Conway et al., 2018; Wadham et al., 2016) or formal (Chenoweth et al., 2019).

Personhood, a person-centred approach to dementia care originally coined by Kitwood (1997), emphasises the need for high-quality interpersonal care and communication, and is dependent on the building and nurturing of mutually respectful, empathic and trusting formal care relationships (Brooker & Latham, 2016; Fazio et al., 2018). Within this framework, people with dementia are recognised as unique individuals who, like all people, need to experience warmth, comfort, attachment, inclusion, meaningful occupation, and connection to self-identity (Kitwood, 1997). More recently, focus has shifted towards these human needs within informal care relationships (Hennelly & O’Shea, 2022). Models described as couplehood (Hellström et al., 2005) and family-centredness (Hao & Ruggiano, 2020) seek to address the unique needs of people with dementia and their informal care partners, including meaningful reciprocity, reminiscence, and ongoing shared experiences. These person-centred care approaches closely align with music therapy practices in addressing the complex needs of people living with dementia (Kelly et al., 2023).

Music therapy offers personal and relational benefits for people with dementia and their care partners and could help to fill this gap in services within formal (McDermott et al., 2013) and informal contexts (Clark et al., 2018; Kelly, 2023; Molyneaux et al., 2022; Thompson et al., 2021a). The psychosocial model of music in dementia (McDermott et al., 2014) recognises how the impact of music on the personal psychology of people with dementia and the social psychology of their environment meets person-centred and relational needs. Community music therapy is also relevant to practice with people with dementia and their care partners as it honours belonging, inclusivity, flourishing, and contribution within a relational social experience (Stige & Aarø, 2012; Stige et al., 2016). More recently, the person-centred music therapy model has identified numerous specific applications used by music therapists to address psychological needs of people with dementia recognised by Kitwood (1997): celebration, collaboration, giving, creation, facilitation, holding, negotiation, play, recognition, relaxation, timalation, and validation (Kelly et al., 2023). Within these music therapy models, participants are afforded the dignity of risk (Woolford et al., 2020) and acknowledged as experts in their own lives. Further, music therapy, as a forum for self-expression, can become a vehicle for exploring and addressing social stereotypes and stigmas (Gross, 2023).

Therapeutic songwriting is a specific method facilitated by music therapists that guides participants to explore self-identity, relationships, personal and shared history, and gain new perspectives (Aasgaard & Blichfeldt Ærø, 2016; Baker, 2016). Baker (2016) defines therapeutic songwriting as “the process of creating, notating, and/or recording lyrics and music by the clients and therapist within a therapeutic relationship to address psychosocial, emotional, cognitive and communication needs of the client” (p.14). Various songwriting methods are described for use with individuals and groups, ranging from lyric substitution within existing songs, improvised creations, and original compositions (Aasgaard & Blichfeldt Ærø, 2016; Baker, 2016). Within a group setting, the songwriting process offers additional benefits, including a sense of commonality, cohesion, belonging, connection, and the development of empathic relationships (Baker, 2016). Group songwriting guided by person-centred, psychosocial and community music therapy models (Baker, 2016; McDermott et al., 2014; Stige & Aarø, 2012) therefore offers a potential space where people with dementia and their care partners can work together to address both personal and relational needs in new ways.

Songwriting research has explored the experiences of people with dementia and their care partners in various group configurations, including with people with dementia only, care partners only, or together as a duo. For people with dementia, songwriting was experienced as a cognitively stimulating process that challenged assumptions about capacity and engagement by shifting the predominant focus from deficits to strengths and resources (Baker & Stretton-Smith, 2018; Hong & Choi, 2011). Care partner only songwriting groups have been described as an empowering experience where participants were able to honestly voice their experiences, feel heard, and gain insight into their carer journey (Baker et al., 2018; Baker & Yeates, 2018; García-Valverde et al., 2022). Clark et al.’s (2021) research involving people with dementia and care partners participating together as duos found songwriting to be a unique social space for storytelling and exploration of shared lived experiences with others in a similar situation, leading to a greater sense of connection with each other, as well as feelings of achievement and the development of new friendships. This previous research recognises group songwriting as a creative, motivating, engaging, social and rewarding endeavour for people with dementia (Baker & Stretton-Smith, 2018; Hong & Choi, 2011) and their care partners participating separately (Baker et al., 2018; Baker & Yeates, 2018; García-Valverde et al., 2022) and together (Clark et al., 2021). However, limited studies have explored how these in-person songwriting experiences might translate to an online environment.

Following the recent pandemic, systematic reviews have begun to explore the ongoing acceptability and accessibility of telehealth services for people with dementia and their care partners (Sekhon et al., 2021; Yi et al., 2021; Zhang et al., 2023). These reviews suggest that telehealth-based interventions are generally regarded as effective and satisfactory by service providers and recipients, offering a high level of convenience. However, concerns remain about the accessibility of telehealth for people with dementia and their care partners who also experience sensory impairments. Further, technical limitations such as limited services to remote areas (Sekhon et al., 2021) as well as challenges experienced by many older people in manipulating technology independently can limit telehealth accessibility (Yi et al., 2021). Nonetheless, overall conclusions suggest that telehealth is a promising, feasible, cost-effective, and acceptable mode of health care delivery for older people with various health conditions (Zhang et al., 2023).

More specifically, benefits and barriers have also been highlighted in telehealth music therapy services, creating a strong argument for ongoing research in this area (Baker & Tamplin, 2021; Clements-Cortés et al., 2023; Knott & Block, 2020; McFerran et al., 2022; McLellan, 2021). Krout et al.’s (2010) early research highlighted how songwriting ideas can be effectively shared online. However, developing therapeutic relationships may take more time than for in-person therapy, and technological limitations, such as audio delay and poor online resolution can detract from the therapeutic experience (Krout et al., 2010). More recently, research has explored the feasibility of online music therapy programs incorporating songwriting with other methods (such as singing familiar songs, music listening, instrument playing, and moving to music) for people with dementia and their care partners (Kelly, 2023; McMahon et al., 2023; Stedje et al., 2023). While these programs acknowledged the online format as an accessible and convenient alternative to in-person music therapy, technology was experienced as a barrier by some participants. Ongoing music therapy research exploring the implementation of telehealth with people with dementia and their care partners is needed to articulate how programs can support engagement, minimise barriers, and deliver optimal therapeutic benefit (Baker & Tamplin, 2021; Wilhelm & Wilhelm, 2022).

Previous music therapy research has specifically explored in-person therapeutic songwriting with people with dementia and care partners participating together (Clark et al., 2020, 2021) and online programs incorporating songwriting and other music therapy methods with people with dementia and their care partners (Kelly, 2023; McMahon et al., 2023; Stedje et al., 2023). However, there is a gap in the research focusing on therapeutic songwriting with people with dementia and their care partners participating as a duo. Therefore, the current study aimed to explore how people living with dementia and their care partners experienced an online therapeutic songwriting program.

## Method

This qualitative study took place as part of a single-group pre-post feasibility study examining online therapeutic songwriting (hereafter labelled ‘songwriting’) involving people with dementia and their care partners (herein referred to as duos). A separate publication is planned to report results from analysis of pre-post data. Ethics approval was obtained by the University of Melbourne Human Research Ethics Committee (Ethics ID: 2022-23220-24871-3). Informed signed consent was provided by all participants or legally designated proxies of participants with dementia (not their co-participating care partner) prior to participation.

### Participants

Participants were recruited through established partnerships with industry and community organisations offering formal support for people with dementia. Interested people were screened for eligibility and invited to participate following an online meeting or telephone call with the program facilitator (Lawson, Kathleen). Eligibility criteria required participants to be 18 years or older, have functional hearing (with or without aids), English communication skills (current or previous if non-speaking), and access to videoconferencing equipment and technology. People with dementia required a formal diagnosis recognised by their affiliated organisation. To understand how online therapeutic songwriting was experienced by people with dementia and their care partners living in both informal and formal contexts and to align with our inclusive approach, recognising that some people with dementia may not be able to participate with an informal care partner, duos were eligible whether living together in the community or separately because the person with dementia resided in a care home (Molyneaux et al., 2022). Thus, care partners could be a spouse/partner, adult child, other relative, friend or formal carer of the person with dementia.

### Theoretical approach and program description

**Figure 1.**
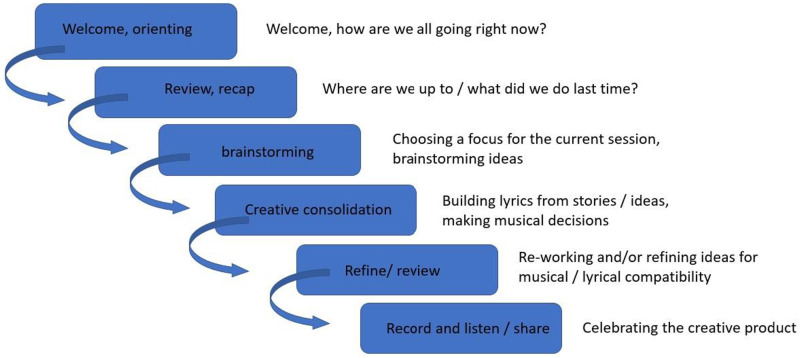
Session flow.

The online therapeutic songwriting program and research design was informed by person-centred songwriting (Baker, 2016), the psychosocial model of music in dementia (McDermott et al., 2014), and community music therapy (Stige & Aarø, 2012). Drawing on these approaches, the program was sensitively facilitated and adapted to meet the unique needs of individuals, duos and groups, supporting participants’ exploration of interests and lived experience throughout the creative process. A range of therapeutic songwriting methods were utilised to facilitate a dynamic process, including song parody, song collage, prose set to music, ‘mini-opera’ and original songwriting (Baker, 2016). While our approach encompassed flexibility to meet the needs of participants in the moment, sessions were also scaffolded using a protocol to provide a level of consistency (Figure 1).

All sessions were facilitated by the same qualified music therapist (Lawson, Kathleen). The program comprised of four duo sessions (phase 1) followed by six group sessions involving two to four duos (phase 2). A two-phase approach was adopted to build rapport with the therapist and familiarity with the songwriting process and online context over the initial sessions. This introduction phase facilitated the transition into groups, which involved added complexities including the development of multiple new relationships and negotiating group dynamics. Duos were allocated to groups based on their care relationship, either informal or formal. Groups included a maximum 4 duos to support individual participation and foster connection between participants (Clark et al., 2021).

Sessions took place using Zoom™ and Aeons™ videoconferencing platforms. Equipment and applications used by the facilitator during sessions and to record songs included a guitar, keyboard, computer, microphone, Spotify™, YouTube™, Garageband™, and Zoom™ whiteboard, as well as song printouts and recordings to assist participants to recall content from the previous session. Participants also used their own instruments where available.

### Data collection and analysis

Aligning with our theoretical approach (Baker, 2016; McDermott et al., 2014; Stige & Aarø, 2012), each duo was invited to share their experiences and perspectives separately in a post-program online semi-structured interview with the music therapist facilitator (Lawson, Kathleen). The interviews were recorded using teleconferencing audio and transcription functions. We drew on Thompson, et al.’s (2021b) guidelines for making qualitative interviews in music therapy research accessible for people with dementia including: existing familiarity and rapport with the interviewer (Lawson, Kathleen); conducted interviews at participant selected times and locations and used pragmatic elements such as songs created during the program and visual prompts; provided questions and information before the interview; offered duo or care partner only interviews as appropriate; and monitored ongoing consent of the person with dementia during the interview. Interview questions asked participants to describe their experiences of the program as a duo and in the group, including any moments that stood out for them, how they experienced the online context, if the songwriting program was similar or different to other support services/groups they attended, and whether they would recommend any changes.

Data were analysed using an inductive, realist-contextualist approach to reflexive thematic analysis (Braun & Clarke, 2006, 2022). Thematic analysis was selected for its relevance to our inclusive theoretical approach, and since research in this area was limited, as a useful method for generating initial insights and social interpretations (Braun & Clarke, 2006). Recognising reflexive engagement with the data as a conscious and intentional process (Jackson, 2016), the following composite epoche was developed to explain how each author was positioned and to identify potential assumptions and preconceptions:

Each author brought a different perspective to the analysis, often leading to robust discussion as themes and subthemes were identified and developed. Clark, Imogen was influenced by the active construction of the project and development of the research questions, based on prior research. Lawson, Kathleen served a dual role as facilitator and researcher and was influenced by their relationships and connections with the participants. Christopher, Neha carried their creative arts experience as a dance therapist, but without direct interaction with participants or music therapy protocols. Stretton-Smith, Phoebe brought experience as a music therapist who had facilitated and researched similar in-person songwriting sessions, but without direct contact to participants in the current project. These varied perspectives delivered a nuanced focus of attention and curiosity to elements of the data, influencing how it was viewed by each author. Consistent commitment to the reflexive process was paramount throughout analysis, as differing impressions amongst the authors were challenged and discussed, with outcomes reached by consensus.

**Figure 2.**
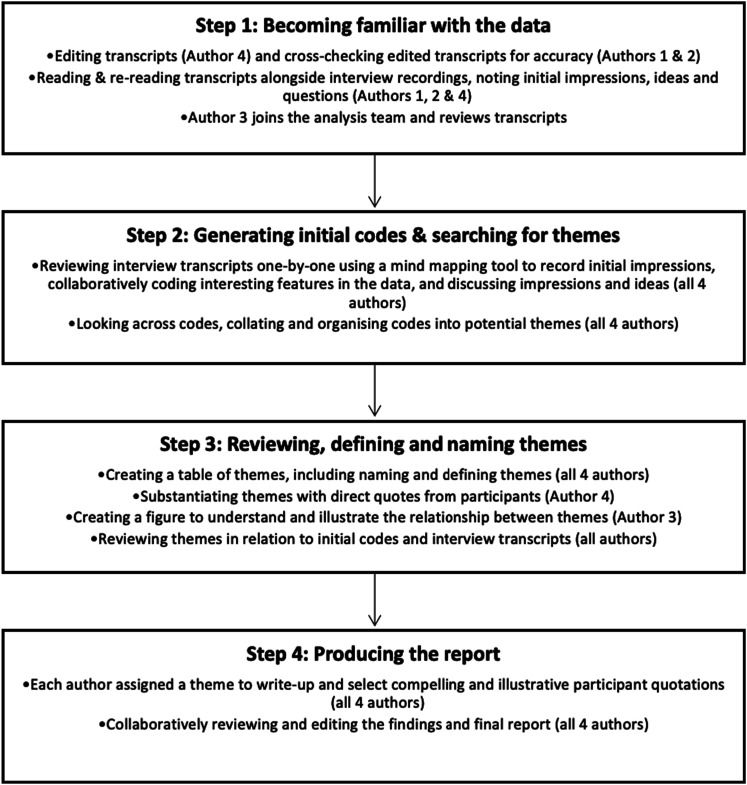
Development of themes.

To begin the data analysis process, Lawson, Kathleen reviewed interview transcripts generated by artificial intelligence alongside the original audio recordings. Clark, Imogen and Christopher, Neha then cross-checked the transcripts for accuracy. During this process, authors familiarised themselves with the data and recorded initial impressions, notes, thoughts, questions, and ideas. All four authors then met online on four occasions to collaboratively review each interview. Codes and initial themes were identified and organised using a mind-mapping tool. The authors met online on a further three occasions to discuss, review, and refine themes. A shared document and printed copies of the mind maps were used to cross-reference and ensure all threads were captured, and themes were arranged alongside subthemes with corresponding codes. Authors were then assigned a single theme to review and ensure data were intrinsically relevant and directly derived from the raw data before drafting a narrative. Finally, the authors worked together to locate overlapping data, establish final themes, and write-up findings. Figure 2 illustrates how the authors collaboratively engaged with the interview data, following the steps of thematic analysis.

## Findings

Nine people living with dementia and six care partners (nine duos) were recruited and participated in the program (Table 1). Five duos were spousal couples living together at home (groups 1 and 2). Four further participants with dementia took part in the program with a formal carer at a dementia care day centre (group 3). Participants lived in metropolitan, regional, and rural areas across two states. Two participants identified as musicians, several described music as a hobby, and all explained how they enjoyed music engagement as a pastime.

**Table 1.** Participant characteristics.

Characteristics	People with dementia (*n* = 9)	Care partners (*n* = 6)
Female/male	7/2	3/3
Age in years^a^	76 (12), 53–90	59 (20), 28–84
Country of birth, *n*		
- Australia	7	5
- New Zealand	1	-
- Italy	1	-
- USA	-	1
Current work, *n*		
- Full-time	-	2
- Part-time	1	-
- Not working	8	4
Location, *n*		
- Metropolitan	5	2
- Regional	3	3
- Rural	1	1
Diagnosis, *n*		
- Vascular dementia	2	-
- Alzheimer’s disease (AD)	2	-
- Younger onset dementia (AD, PPA)	3	-
- Not known	2	-

*Note.* AD, Alzheimer’s Disease; PPA, Primary progressive aphasia.

^a^Mean (SD), Range.

Songwriting sessions ranged from 26 to 63 mins (M = 45 mins) depending on participant engagement and energy levels. Participants attended an average of four duo sessions and three group sessions. Altogether, participants created 36 songs during the program, primarily through methods of song parody and original songwriting (Table 2).

**Table 2.** Online therapeutic songwriting groups.

Duo names	Song topics	Songwriting methods	Sessions attended
Group 1 (spousal)			
Sharon and chris^a^	Family, relationship/shared experiences, Sharon’s childhood, birth of first child	SP	4 duo, 6 group^b^
Carlo and louise^a^	Daily routine, holiday reminiscing, how they met, Carlo’s childhood	SP, OS, SC	4 duo, 6 group^b^
Glenda and John^a^	Shared daily life and pets, activities of that day	SP, OS	3 duo, 1 group
	Group topics: Introductions and songwriting experiences, travel memories, living with dementia and how their lives are changing	SP, SC	
Group 2 (spousal)			
Sylvia and Justin^a^	Family, holidays, shopping, feeling unwell, first home, living with dementia	SP, OS	10 duo, 0 group^b^
Mark and laura^a^	Birdwatching hobby, homes they’ve lived in, nursery-rhyme for granddaughter	SP, OS	3 duo, 0 group^d^
	Group topics: No group songs		
Group 3 (formal)			
Valerie and Eliza^c^	‘Going with the flow’, special memories, memory challenges, favourite things to do	OS, SP	4 duo, 6 group^b^
Margot and Eliza^c^	Exploring self-identity, reminiscence and recounting of Margot’s life journey	OS	3 duo, 5 group^b^
Kitty and Eliza^c^	‘Silly song’ derived from immediate environment, Kitty’s in-the-moment ideas	OS	1 duo, 6 group^b^
Julia and Eliza^c^	Julia’s history, childhood, frustration/challenges, feelings, memories, relationships	SP, OS	4 duo, 0 group^d^
	Group topics: Coming together to write a song, events and relationships between participants, memory and challenges, mutual support and using humour to ‘get through’, individual hopes, fears and facing an unknown future with positivity	OS	

*Note.* SP, song parody; OS, original songwriting; SC, song collage.

^a^Spousal care partner.

^b^Duo participated in interview.

^c^Formal care partner.

^d^Care partner participated alone in interview.

Six people with dementia and five care partners agreed to participate in post-program interviews. Interviews ranged from 18 to 37 mins (M = 22 min). The formal caregiver participated in four separate interviews either with their duo partner with dementia (*n* = 3) or alone (*n* = 1) because the participant with dementia had died following the four duo sessions. One duo withdrew from the program after three duo and one group session owing to a sense of overwhelm and reduced flexibility within the online group setting and were not interviewed. Another duo only attended 3 duo sessions and the care partner participated alone in the interview as the person with dementia became unwell.

### Themes

**Figure 3.**
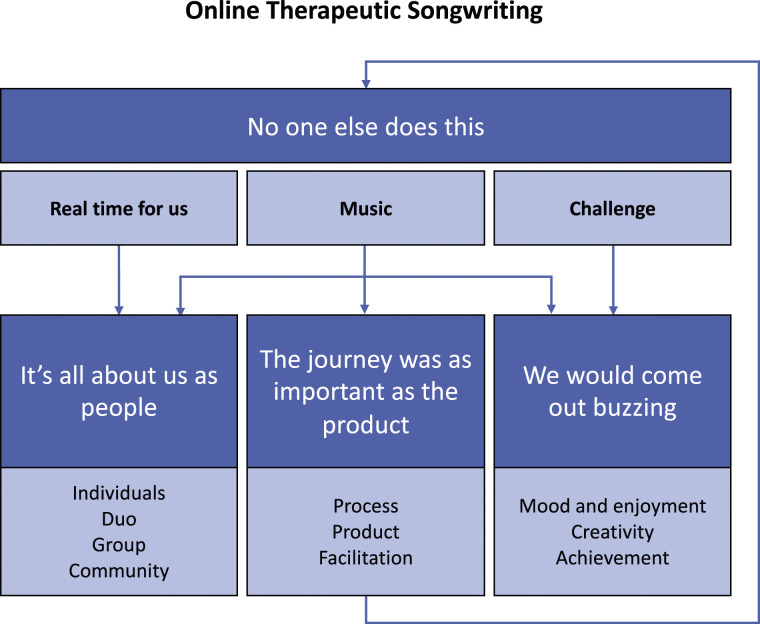
Themes describing participants’ experiences of online therapeutic songwriting.

Four themes were developed and titled using individual participant quotes representing group data as follows: (1) No one else does this; (2) It’s all about us as people; (3) After the sessions we would come out buzzing; and (4) The journey was as important as the product. Main themes and sub-themes are illustrated in Figure 3 and described in the following narrative with participant quotations. All participants are referred to by pseudonyms and care partners are marked as spousal* or formal^#^.

#### Theme 1: No one else does this (Justin*, Chris*, Laura*)

The songwriting program was a unique opportunity with positive challenges, musical engagement, and new ways for duos to connect.

##### No one else…challenges us in this way (Justin*)

Participants described songwriting as cognitively stimulating, noting, “to fit words into a line of a song, you’ve got to use your brain” (Justin*). This was differentiated from other services, which *“have less of a push on any of Mark’s intellectual capacities”.* Participants expressed feeling well-supported to engage in this “new” (Louise*) challenge despite apprehensions: “I was a bit reticent at the beginning. I wasn’t too sure…how it was going to pan out, particularly with Sylvia’s involvement” (Justin*).

Cooperation and collaboration were part of the unique challenge. Groups included “people with word-finding difficulties and aphasia, and then others that [were] actually able to express themselves…quite well. So [it]…required a bit of give and take” (Chris*). However, participants valued this challenge: “the actual result...was the...participation of the three of us in trying to achieve something that was...difficult (Justin*).

##### No one else...does music (Justin*, Laura*)

Participants explained how the novel nature of songwriting “played to [their] strengths” (Louise*), offering a space to engage in something they mutually enjoyed: “We both like music and enjoy listening...It was good, you know, we didn’t diverge. We were all about the music” (Louise*). For some, it was a unique opportunity to reconnect with their musical identity. Chris* described how the program *“hearkened back for me, back to my singing career”,* while Laura* highlighted how *“no one else focuses on music and music’s such a huge part of [Mark’s] life”.*

##### No one else...creates real time for us (Sharon)

Songwriting created “*real time”* for duos through opportunities for deep personal and shared engagement. Care partners noted how this was different to normal: “*I don’t get to be with them like this...it’s amazing and I love it” (Eliza*^*#*^*).* Care partners also reported feeling ‘seen’ as individuals and partners, rather than just as “a conduit between the person with dementia and [their] services” (Chris*).

“Nothing is focussed on the carer or the couple as a whole...this actually had benefit for both of us because it talks about our lived experience...It enabled us to have time out to reflect on those things and appreciate them” (Chris*).

#### Theme 2: “It’s all about us” as people (Sharon, Carlo)

Participants highlighted the focus on them as people at individual, duo, and group levels, as well as within their community.

##### It’s all about us…as individuals

Participants emphasised the *“sincerity and honesty*” (Chris*) involved in the songwriting process as they connected with their personal identity, significant memories, and life experiences: “There was one about ‘You must remember this, when we first shared a kiss’...one of the early times (Chris*). Oh yeah, that’s right (laughs)” (Sharon). For some, personalising their song lyrics was important: *“That’s the music – our family, the children of all. I like it, it goes, 1 2 1 2…yes, and then [son’s name] …Oh yes!” (Sylvia).*

##### It’s all about us…as a duo

Participants recognised the program as a “*dually inclusive*” (Louise*) experience that drew on their “lived experiences and the ability to work together” (Chris*), highlighting their existing relationship as a resource. Furthermore, many participants described how the songwriting process “actually helped our relationship” (Chris*). Songwriting supported the formal caring relationship: *“It’s brought us closer together and I think she trusts me a bit more now because she can see I’m trustworthy” (Eliza*^*#*^*)*. Similarly, spousal couples described the importance of sharing the experience, which promoted *“quality time together”* and “reinforce[d] the reasons why we are together” (Chris*).

“We don’t often go down memory lane, we don’t contemplate on one aspect of your relationship for a little bit” (Louise*).

##### It’s all about us…as a group

Group sessions offered an opportunity to meet and interact with others. Participants described how the heterogeneity of the group made it interesting, resulting in song creations that were “*a bit of everyone” (kitty).*

“The songs that other people had created, sharing those were just totally different, and also the input from people was different in the song, the song choices and their experiences...so it was interesting reflecting on that, and seeing how things are for different couples” (Chris*).

Group members developed new relationships and learnt more about “*each other as we put things in [to the song]” (Kitty).*

“Learning about, especially the other people, was just awesome…learning our likes and dislikes…what music we like and what we don’t like, and what we choose…everyone was so different” (Eliza^#^).

Couples also reflected on how these new relationships helped them to realise they were not alone.

“Meeting and bonding with other carers and people with dementia…especially for Sharon to realise that she’s not alone, and that there are other people...going through similar experiences to what we’re going through. I think [Sharon] really appreciated that” (Chris*).

Contrastive perspectives were shared about duo and group sessions. Some felt the group was “*more interesting, more fun [and] less nerve wracking [with] more ideas”* and “*not as much stress on you” (Kitty).* Participants valued the joint engagement and shared focus, being *“all together, all laughing at the same things” (Eliza*^*#*^*).* However, co-creating a song in a group was also experienced as “*a little bit vulnerable” (Margot)* compared to duo sessions which “*didn’t seem as vulnerable” (Margot)* and discussion was “*more free” (Eliza*^*#*^*).* Importantly, participants acknowledged “everyone’s journey is different” (Louise*) and the group setting “wasn’t for everyone” (Louise*).

##### It’s all about us…as a larger community

Participants described friendships developed in the program “as a side benefit”: “It’ll be really nice to...follow up from there, and maybe continue friendships and be able to support each other” (Chris*).

Participants also spoke about the program in the broader context of their “dementia journey” (Louise*), sharing their experience with family, friends, and community:

“It’s something to talk about with friends, it’s going out on another limb on a tree that we haven’t been before…that’s what I found really gratifying...we do lots of things around dementia, and this is another thing that we access, that we did together…so, it’s all part of the learning. And if our little part of jigsaw can help research, then…” (Louise*)

Finally, some participants acknowledged the social stigma associated with dementia and how songwriting was a unique positive experience:

“There’s a lot of…negativity and stigma attached with what’s happening, and there’s a lot of misconceptions with people around us…But this is...an experience that has been positive…for the both of us. And to be honest, we haven’t been able to have or replicate those sort of experiences” (Chris*).

#### Theme 3: After the sessions we would come out buzzing (Chris*)

Participants described the sessions to be enjoyable, fun, creative, mood enhancing and leading to a sense of achievement.

##### I enjoyed every moment, everything was fun (Carlo)

Experiences of joy were emphasised by multiple duos: *“The best bits would just be…having fun and laughing. And making the songs that we have forever…honestly, I had so much fun” (Eliza*^*#*^*).* Further, some care partners described joy from witnessing their partner’s enjoyment: “I think the overall standout was Mark smiling away...it brought him a lot of joy, and of course that brings me joy!” (Laura*).

##### When we’re feeling a little bit tired and totally down…the happy chemicals...are released (Chris*)

Participants’ experiences of enjoyment and fun were not momentary emotions but left them feeling “refreshed and ready to do other activities and move on…feeling better about myself and about our relationship” (Chris*). Group members identified specific aspects of the program that helped lift their mood, including singing and songwriting: *“Sometimes, if we weren’t having the best day...we wrote a song about it…and that got us through” (Eliza*^*#*^*)*.

The songwriting program also offered some respite to “*forget about all that negative stuff and focus on something totally different” (Sharon).* Participants emphasised how getting in touch with their creative side through choice-making, spontaneity, and self–expression to “*come up with songs…was honestly amazing” (Eliza*^*#*^*).*

##### Songwriting led to a real sense of achievement (Justin*)

Multiple participants described “a real sense of achievement from creating something, even if it’s just for us” (Chris*). Participants linked this to multiple facets of the experience, including creating songs that were “*true,” “authentic”* and “good quality” (Chris*), describing “the result [as] quite incredible” (Justin*).

The novel nature of the program expanded participants’ understandings of what songwriting and making music could encompass. “I was pretty blown away by the fact that we could put his grenade story to an opera. When you talk about making music…I hadn’t thought about that at all” (Louise*).

Participants voiced feelings of surprise and accomplishment about their contributions, which surpassed expectations:

“I’m actually very proud because...I didn’t know that I could do this” (Margot).

“As I listen to it, I think, ‘oh that girl’s got some brains’” (Kitty).

#### Theme 4: The journey was as important as the product (Louise*)

Participants equally valued both the songwriting process and the song products, recognising how skilful, sensitive facilitation guided positive experiences and prompted investment in the program.

##### You’ve got something at the end of it (Justin*)

Song creations representing special life events and meaningful associations were described as a valuable resource, legacy, and “*treasure*” (Laura*). One participant experienced *“a feeling [of having] done something, and that it could be a good future” (Carlo)*, suggesting the song offered a sense of hope. Participants also spoke about reconnecting with music beyond sessions as they “*listened to [their songs]…[brought out] the guitar, and … read the lyrics*” (Louise*) with some care partners explaining how songs were used to prompt memory:

“When I play the music then he’s like, ‘Oh, yes’…he remembers at that point…He particularly likes the Salisbury Hill one...it’s the most familiar...or the most catchy, perhaps” (Laura*).

##### I loved the songs at the end of it… but if we had just had our sessions, I would have been happy with that (Louise*)

While participants recognised the importance of their songs, they explained how the process of writing them was also rewarding and offered opportunities for “chats, challenges” (Justin*) and “learning” (Louise*, Sharon, Kitty). Participants spoke positively of their investment as they “*‘look[ed] forward to having that time”’ (Sharon)*, and *“made it work” (Eliza*^*#*^*)*. Participants were “*amazed”* with *“what [they] came up with… because it was all online” (Eliza*^#^), facilitating creativity in their own homes: “He brought this incredible musical talent to our table, to our kitchen table” (Louise*).

##### Facilitation… flexibility, great care and body language (Chris*)

Despite not having “any expectation” (Justin*), participants recognised how the facilitator’s efforts “*made the sessions interesting”* (Sharon) and “led…to a good result” (Justin*, Carlo). Participants further commented on the facilitator’s ability to *“read the situation”* and adjust accordingly with clear, sensitive, and respectful communication *“even* via zoom” (Laura*).

“You engaged Mark…in normal adult conversation, but…you actually slowed your speech enough and you were willing to repeat things so that the language side of things was given to him as well” (Laura*).

Participants commented on how the facilitator’s creative strategies promoted engagement: “fill[ing] gaps when people may be having a little bit of difficulty…prompting, but not actually taking over…it was really great the way you complemented peoples’ ideas” (Chris*). “Yeah” (Sharon). Participants further explained how “other [services] are not trained in doing things the way [the facilitator was]” (Justin*). This sensitivity and skilful expertise made it “easier, having a facilitator there than us trying to talk to each other, because there are subjects that we wouldn’t necessarily broach” (Chris*).

This sensitive facilitation mitigated inherent challenges of online engagement experienced by participants. These challenges included “internet lag” (Chris*, Eliza^*#*^*)* and “freezing” (Louise*), which disrupted the continuity of sessions. Some felt embarrassed or upset about their own digital literacy: “You do feel you’re letting people down…here I go again, it’s not working” (Louise*). Others noted “s*ongwriting [as an online activity] could be wordy*” (Sharon), and “if there’s something else happening out the window…Mark very quickly just gets distracted” (Laura*). Nonetheless, some carers were surprised that their partners remained engaged: “And it wouldn’t have surprised me if she had sort of walked off halfway through sessions…but you [talking to Sylvia] stick with it…which was good” (Justin*). “Yes” (Sylvia).

## Discussion

This project explored how people with dementia and their care partners experienced an online therapeutic songwriting program. The online context overcame geographical barriers, enabling duos from remote regional and rural areas to access and participate in the program. Further, the project extended previous in-person songwriting research with duos (Clark et al., 2020, 2021) by including participants from spousal and client/formal carer relationships in duo (phase 1) and group sessions (phase 2).

Our analysis revealed complex interconnections between the four main themes and within the subthemes of each theme (Figure 3). ‘*No one else does this’* (Theme 1) was identified as overarching, encompassing participants’ experience of online songwriting as significantly different to other programs and services. The songwriting program offered a unique combination of ‘real time’ for participants to meaningfully engage with each other, with music, and to be challenged in ways that were welcomed. Participants endorsed this ‘real time’ with focus on them as people – as individuals, duos, groups, and contributors within their community – as a space to explore capacity and skills, lived experience and relationships from a perspective not dominated by a diagnosis, and not offered elsewhere (Theme 2). The challenge of creating songs and the resulting products sparked feelings of enjoyment, enhanced mood, and achievement (Theme 3). Finally, the sensitively facilitated songwriting journey within an online environment was perceived by participants as being as important as the song products (Theme 4).

In recognising that ‘no one else does this’ (Theme 1), participants differentiated the online songwriting program from other services. Consistent with previous songwriting research, our program was experienced as a unique opportunity to engage in a creative process, reconnect with music, and take on positive creative and cognitive challenges (Baker et al., 2018; Baker & Stretton-Smith, 2018; Clark et al., 2021; García-Valverde et al., 2022). Our findings also recognised therapeutic songwriting as a space that gave care partners a voice and forum for expression, thereby addressing an important gap in services (Baker et al., 2018; Baker & Yeates, 2018). Further, both spousal and formal care partners in our study reflected that they did not get to spend time with the person with dementia ‘like this’ in their regular interactions, aligning with prior music therapy research in formal care settings (Lee et al., 2022). Like Clark et al., 2021, this project offered sessions attended by both participants with dementia and their caregivers together in groups but differed with the inclusion of duo sessions as well. Our findings add to previous research in suggesting that unique benefits from songwriting can be experienced by duos in individualised sessions as well as across various group configurations including for people with dementia (Baker & Stretton-Smith, 2018; Hong & Choi, 2011), care partners (Baker et al., 2018; García-Valverde et al., 2022), or together in groups (Clark et al., 2021).

Focussing on personal identity and close relationships, participants explained how the songwriting program was “all about [them] as people” (Theme 2). These findings align with theoretical orientations underpinning the project, including person-centred songwriting approaches (Baker, 2016), the psychosocial model of dementia care (McDermott et al., 2014), and community music therapy (Stige & Aarø, 2012), acknowledging participants as experts in their own lives. Within this program, participants described how different ways of engaging helped them to build trust and learn more about each other (in formal care relationships) and reinforced the reasons why they were together (for spousal duos). Participants recognised their relationships as a valuable resource, which can be overshadowed by everyday stressors (Stedje et al., 2023). Participants noted that this unique relational experience challenged their unconscious preconceived notions around capacity. It is possible that this heightened self-awareness and recognition of strengths voiced by people with dementia and their care partners through a song may have a ripple effect in offering insights about lived experiences and relationships and compelling listeners to also reconsider their assumptions (McFerran et al., 2022), thereby addressing a societal call for a shift from deficit-oriented thinking associated with ageing (Fletcher, 2019).

Consistent with previous online music therapy research, collaborative engagement within songwriting groups led to new relationships and provided a sense of understanding, inclusivity, and community with other duos who were in a similar situation (Kelly, 2023; Molyneaux et al., 2022). Further, our findings mirror previous research in recognising group songwriting as a platform for cultivating personal agency among care partners (Baker & Stretton-Smith, 2018; Baker & Yeates, 2018; García-Valverde et al., 2022), and consolidating shared identity within close relationships (Clark et al., 2021). However, while several participants in our project valued the collaborative group context finding it easier to engage as part of a group than in duo sessions, some participants felt more vulnerable. One duo withdrew from the program owing to a sense of overwhelm and reduced flexibility within the online group setting. Another participant with dementia appeared less comfortable to engage in the larger group but maintained their participation at a more subdued level than during duo sessions. Baker and Stretton-Smith (2018) found similar contrasting experiences in recognising benefits such as connection, collaboration, and cohesion within groups, while also highlighting challenges for people with dementia, including feelings of self-consciousness. Future research might explore the affordances and limitations of individual and group therapeutic songwriting, specifically experiences of vulnerability.

Participants described how they would “come out buzzing” after sessions, identifying enjoyment and fun as key elements of their engagement (Theme 3). These experiences suggest that songwriting offered a sense of respite from other pressures, while also contributing to enhanced mood and coping. Consistent with previous research, songwriting can lift mood regardless of whether groups include just care partners (Baker et al., 2018; Baker & Yeates, 2018; García-Valverde et al., 2022), people with dementia (Baker & Stretton-Smith, 2018; Hong & Choi, 2011) or, as in this project, duos participating together (Clark et al., 2021). It is also possible that these fun and enjoyable experiences in songwriting sessions within a supportive therapeutic environment scaffolded the exploration of more challenging moments and topics that participants would not normally “broach” (Chris*) (Baker, 2016).

The cognitive and creative challenge of songwriting reinforced a sense of personal agency, capability, and pride in song creations, challenging self-perceptions among participants with dementia and their care partners. This experience of achievement helped participants to think differently about themselves. For example, one participant with dementia exclaimed, “this girl’s got brains” (Kitty). Further, one group wrote a song together discussing their experience of the day-to-day aspects of living with dementia, framing it around moments of hope and success, while also acknowledging the changing nature of their circumstances and the incumbent social impacts. These findings mirror Baker and Stretton-Smith’s (2018) research involving people with dementia in highlighting how the songwriting process was a unique forum for challenging social stigmas and consolidating positive self-concept. Since opportunities for people with dementia and their care partners to express themselves, be heard, and flourish are not easily accessible (Francis & Hanna, 2022), songwriting may fill an important gap in current services.

Participants explained that the journey of writing a song with guidance from a sensitive facilitator was “as important as the product,” (Louise*) (Theme 4). These findings demonstrate the importance of having a qualified facilitator to maintain an empathic and non-judgemental space where participants could explore ideas, themes, experiences and feelings with vulnerability and authenticity. This sensitive and ethically minded facilitation, which is intrinsic to music therapy (Stedje et al., 2023; Trondalen, 2023) may have afforded participants the dignity of risk to engage with emotionally and cognitively challenging concepts, ideas, and experiences (Marsh & Kelly, 2018).

The online program enabled participants to join sessions from a familiar and comfortable environment, which may have encouraged engagement, particularly among participants with dementia who can feel disorientated in new contexts (Lopez et al., 2018). The facilitator was also able to draw on prompts in the duo’s environment to support creativity and stimulate discussion. Conversely, some participants felt online engagement was challenging over a 2-dimensional screen. These findings suggest that a balance between engagement and the convenience and familiarity of an online environment needs to be considered when deciding whether individuals will benefit from telehealth music therapy (Baker & Tamplin, 2021).

Similar to other online music therapy programs, participants were able to join the project from remote locations overcoming logistical barriers such as transport and access (Kelly, 2023; McMahon et al., 2023; Molyneaux et al., 2022; Stedje et al., 2023). However, we recognised an ethical dilemma, as not all people with dementia and their carers have access to the technology or the digital literacy required to independently take part in an online program. Another ethical dilemma was raised when we considered additional burdens that may be placed on participants to manage both their own emotional wellbeing and that of their partner without the physical presence of a music therapist. In this study, we offered songwriting within a formal dementia care day centre to support safe and emotionally contained online engagemethin familiar environments or through community service collaborations, which can be tailored to meet the individual needs of participants.

A limitation of the current study was our use of ‘text only’ interview transcripts and thematic analysis, which may not have captured nuances in speech and non-verbal communication. This limitation could be addressed with interpretative phenomenological analysis (IPA) as used in previous songwriting research with people with dementia (Baker & Stretton-Smith, 2018; Clark et al., 2021). IPA allows researchers to explore interview data through an idiographic lens, recognising nuances in communication, such as body language and vocal tone (Smith et al., 2009). Moreover, ethnographic (Ercole, 2022; Procter, 2013) and arts-based (Manji et al., 2023; Moss & O’Neill, 2017) methods utilising various data such as facilitator session descriptions, video footage, lyric contents and facilitator experiences, as well as interview data might better illustrate authentic experiences of participants.

This project included both formal and informal care partners, which has rarely been explored in music therapy research and could be considered a limitation. However, similar to Molyneaux et al. (2022), we wanted to provide an inclusive program recognising that some people with dementia would not be able to participate with an informal care partner. We also wanted to explore the experiences of people with diverse backgrounds and perspectives. Further, we were mindful of informal and formal care partner voices during the data analysis process and believe emergent themes captured nuances across the different care relationships without compromising findings. It is also worth noting that the formal care partner, Eliza, participated in four duos and corresponding interviews. While potentially a limitation, the self-reported benefit of Eliza’s engagement was an extended opportunity to learn more about her duo partners with dementia through the songwriting process. The enhanced relationships that resulted from the program enabled us to work with Eliza during interviews to support contributions from her duo partners. During the analysis phase, we were also conscious of Eliza’s involvement in four interviews and ensured that her contributions were balanced across the full participant group.

A further potential limitation could be the dual role of Lawson, Kathleen in facilitating sessions, conducting interviews, and analysing data. It is possible that preconceived perspectives may have influenced both the participants’ and interviewer responses, and Lawson, Kathleen impressions of the data. However, we applied particular attention to Lawson, Kathleen contributions in reviewing the interview transcripts and during the analysis process through robust discussion. In alignment with our epoche, this process was used to ensure that any interpretations came directly from the interview data rather than from Lawson, Kathleen assumptions, memory, or impressions. Further, in keeping with recent research, our findings suggested that Lawson, Kathleen familiarity and established rapport fostered comfort in participants with dementia while also helping the interviewer to prepare and provide appropriate support and prompts (Thompson et al., 2021b).

This project extended previous therapeutic songwriting research by exploring experiences of people with dementia and their care partners who participated in online songwriting, both as a duo and in a group. Our findings open the potential for future culturally diverse programs and globally based research, which could be offered more broadly to serve those who cannot attend in-person sessions. Future research and songwriting programs might draw on the findings to explore a more tailored approach to songwriting. For example, some participants in our project felt more vulnerable in groups while others felt stimulated. Some participants felt the online context improved accessibility whereas for others it was challenging. Further, while some participants in the current project found the online song recording session challenging, participants in previous in-person songwriting research commented that recording sessions were a highlight (Clark et al., 2021). Recognising these unique needs and experiences, future research might explore how different songwriting program designs are experienced by individuals and develop guidelines that could be used by both participants and music therapists. Using an arts-based approach, songwriting programs could also be extended with opportunities to share songs more broadly in community events and through social media. Data from these events and social media engagement could be collected from audiences to understand how songs are perceived, possibly promoting the voices and strengths of people with dementia and their care partners, and mitigating negative social stigmas and biases.

In conclusion, our research reinforces the meaning and value of therapeutic songwriting as a space in which people living with dementia can participate and flourish. Despite some challenges, the online songwriting program was engaging, accessible, and enjoyable, and something participants would have continued if it was available. These findings suggest that there are limited similar arts-based opportunities where people with dementia and the care partners can participate together, and further sustainable online songwriting and music therapy programs are warranted.
